# miR-21 induces endothelial progenitor cells proliferation and angiogenesis via targeting FASLG and is a potential prognostic marker in deep venous thrombosis

**DOI:** 10.1186/s12967-019-2015-z

**Published:** 2019-08-15

**Authors:** Xiaolong Du, Lei Hong, Lili Sun, Hongfei Sang, Aiming Qian, Wendong Li, Hao Zhuang, Huoqi Liang, Dandan Song, Chenglong Li, Wenbin Wang, Xiaoqiang Li

**Affiliations:** 1 Department of Vascular Surgery, Nanjing Drum Tower Hospital, The Affiliated Hospital of Nanjing University Medical School, Nanjing, 210000 China; 2 Department of Vascular Surgery, The Second Affiliated Hospital of Soochow University, Suzhou, 215000 China; 3 Department of General Surgery, The Fourth Affiliated Hospital of Anhui Medical University, Hefei, 230032 China

**Keywords:** miR-21, Endothelial progenitor cells, Proliferation, Angiogenesis, Venous thrombosis, Prognosis

## Abstract

**Background:**

Deep venous thrombosis (DVT) of lower extremities is a common thrombotic disease, occurring either in isolation or as a complication of other diseases or procedures. MiR-21 is one of important microRNAs which play critical role in various cellular function. This study aim to determine the effect of miR-21 on endothelial progenitor cells (EPCs) and its role in predicting prognosis of DVT.

**Methods:**

EPCs was isolated from DVT models and control subjects. miR-21 expression was confirmed by RT-PCR. Potential target mRNA was predicted by bioinformatics analysis. EPCs biological functions were examined by CCK-8 and tube formation assay. Besides, miR-21 expression was determined in DVT patients to investigate the correlation between miR-21 expression and prognosis of DVT. Cox proportional hazard regression analyses were also performed to reveal the risk factors associated with prognosis.

**Results:**

Here, we found miR-21 was downregulated in EPCs of DVT model rats. Increased miR-21 expression promoted proliferation and angiogenesis of EPCs. Moreover, we demonstrated that FASLG was a target of miR-21 and revealed that FASLG knockdown inhibited function of EPCs. Upregulation of miR-21 led to thrombus resolution in a rat model of venous thrombosis. In addition, lower expression level of miR-21 in DVT patients was associated with an increase of recurrent DVT and post thrombotic syndrome (PTS). Furthermore, Cox proportional hazard regression analyses demonstrated miR-21 expression level as an independent predictor of recurrence of DVT.

**Conclusions:**

Our data revealed a role of miR-21 in regulating biological function of EPCs and could be a predictor for recurrent DVT or PTS.

**Electronic supplementary material:**

The online version of this article (10.1186/s12967-019-2015-z) contains supplementary material, which is available to authorized users.

## Background

Deep venous thrombosis (DVT) is a global medical problem. The annual number of new cases of DVT worldwide is estimated over 10 million [1], making it one of the most common peripheral vascular diseases. Approximately 20–50% of patients with symptomatic DVT will develop post thrombotic syndrome (PTS) over the next couple of years [2]. Symptoms included chronic pain, intractable edema, skin alterations and leg ulcer. 40% patients with proximal DVT have an associated pulmonary embolism (PE), of which 20% will die before diagnosis due to serious blockage in blood flow in lung [3]. Virchow’s triad indicated three broad categories of factors, including hypercoagulability, hemodynamic changes and vascular endothelial injury, contributing to thrombosis. Endothelial progenitor cells (EPCs) are the precursor cells of endothelial cells. Previous studies have established that EPCs contributed to formation of new blood vessels and tissue vascularization during ischemia [4, 5]. Besides, there is evidence that the number of EPCs reduced in patients with cardiovascular risk factors [6].

MicroRNAs are small non-coding RNA that functions in RNA splicing and post-transcriptional regulation of gene expression [7]. Previous studies have established that microRNAs participated in all the most important processes and regulated all facets of normal and abnormal cellular activity, including the regulation of cellular differentiation, proliferation and apoptosis [8, 9]. MiR-21 has been studied in serval diseases, such as pulmonary fibroblasts [10], breast cancer [11] and non-small cell lung cancer [12]. It is reported miR-21 is related to apoptosis in human umbilical vein endothelial cells (HUVECs) under physical stimuli [13]. Few studies have described the effect of miR-21 on endothelial progenitor cells in deep venous thrombosis. Besides, recent studies demonstrate that miRNAs are present in normal human blood and are comparatively insensitive to RNA degradation, and it has been suggested that miRNA might be instructive and meaningful to predicting prognosis in cancer [14, 15]. At present, study on prognostic markers and the effectiveness of outcome prediction of the miRNA signature in DVT patients is limited.

Here, we found the down-regulation of miR-21 in rat DVT model and reported that the influence of differently expressed miR-21 on EPCs function via targeting FASLG. Furthermore, we identified that lower expression level of miR-21 was an independent predictor of recurrent DVT. Our results provide insight into the role of miRNAs in the regulation of EPCs function and suggest that miR-21 might be a potential prognostic marker in predicting outcome for DVT patients.

## Materials and methods

### Animal models construction

Male Sprague–Dawley (SD) rats (200–250 g) were purchased from Shanghai Laboratory Animal Center (Shanghai, China) and were kept in specific pathogen-free (SPF) animal rooms. All the procedure was approved by the Institutional Animal Care and Use Committee of the Second Affiliated Hospital of Soochow University. This rat model is well described in previous studies [16]. In brief, the rats were anesthetized by intraperitoneal injection of 7% pentobarbital and underwent midline laparotomy in order to dissect the inferior vena cava (IVC) free from aorta. And then IVC was ligated just below the upper renal vein with a 6-0 Prolene sutures. The posterior venous branches were also tightened. After that, the confluence of iliac vein was clamped with vascular clips for 15 min. Then the incision was closed and the rats were allowed to recover after surgery. Thirty rats were randomly divided into three groups (n = 10 for each group): (A) blank control group received 1 ml EGM-2-MV medium, (B) lentivirus vector group received blank vector and (C) miR-21 overexpression group received pGLV3-H1-Puro-miR-21.

### Cell isolation and culture

EPCs isolation was performed as previously described [17]. Briefly, male SD rats (200–250 g) were sacrificed and bone marrow was harvest from femurs and tibias. Mononuclear cells were acquired with density gradient centrifugation and cultured in EGM-2-MV (Lonza, MD, USA) medium at 37 °C in a 5% CO_2_ incubator. Non-adherent cells were washed 4 days after culture. Then medium was changed every 2 days. EPCs in passage 3–4 were used for subsequent experiments.

### Patients samples

Seven milliliters of venous blood were collected from DVT patient from July 2012 to June 2015 in our department. Inclusion criteria consisted mainly age range 30–50 years, suffered from primary acute DVT extending to the high femoral or iliac vein, symptom duration of less than 2 weeks, verified by ultrasound or digital subtraction angiography (DSA), good functional status. The following exclusion criteria were applied: isolated infrapopliteal thrombosis, contraindications to anticoagulation or thrombolytic agents, with malignant tumors, bacterial endocarditis, during pregnancy and declined to provide informed consent. The whole blood was centrifuged at 4 °C, 2000 rpm for 5 min followed by centrifugation at 12,000 rpm for 15 min. The serum samples were portioned in aliquots and stored at − 80 °C. The protocols were approved by the Institutional Review Board of Second Affiliated Hospital of Soochow University. Informed consent was obtained from each participant prior to specimen acquisition.

### Serum RNA isolation

Small RNAs were extracted from 500 μL of serum using a miR-PARIS kit (AM1556) according to the manufacturer’s instructions. To allow for normalization of sample-to-sample variation in RNA isolation, synthetic Caenorhabditis elegans miRNAcel-miR-21 (purchased as a custom RNA oligo nucleotide from Qiagen) was added (50 pmol/L in a 5 μL total volume) to each denatured sample.

### Quantitative real-time PCR (RT-PCR) analysis

Total RNA of EPCs was extracted with Trizol Reagent (Invitrogen; Carlsbad, CA, USA). Mature miRNA expression analysis was done using the miRNA real-time PCR quantitation kit (Applied Biosystems, Foster City, CA, USA). The expression of miR-21 was carried out using the Applied Biosystems 7500 Real Time PCR System, with U6 as an internal control. mRNA expression analysis was performed using Power SYBR Green (Applied Biosystems, Foster City, CA, USA). PCR primers (forward and reverse) were as follows: rno-mir-21, forward: GCGGCGGTAGCTTATCAGACTG and reverse: ATCCAGTGCAGGGTCCGAGG; U6, forward: GCTTCGGCAGCACATATACTAAAAT and reverse: CGCTTCACGAATTTGCGTGTCAT; GAPDH, forward: CGCATCTTCTTGTGCAGTG and reverse: GAGGGTGCAGCGAACTTTATT.

### Cell transfection

To regulate the expression of miR-21 in EPCs, miR-21 agomir, antagomir or respective negative control were transfected into cells with Lipofectamine 3000 (Invitrogen; Carlsbad, CA, USA). 72 h after transfection, cells were harvested for subsequent experiments. Transfection efficacy was evaluated by qRT-PCR. The sequence of miR-21 agomir were: 5′-UAGCUUAUCAGACUGAUGUUGA-3′; agomir negative control were: 5′-UUCUCCGAACGUGUCACGUTT-3′; miR-21 antagomir were: 5′-UCAACAUCAGUCUGAUAAGCUA-3′; antagomir negative control were: 5′-CAGUACUUUUGUGUAGUACAA-3′. The target sequence of siRNA against rat FASLG (NM_012908.1) was 5′-GCAGAACUCCGAGAGUCUATT-3′.

### Proliferation assay

A total of 1 × 10^4^ cells of EPCs were seeded to 24-well plates in a final volume of 800 ul medium for assessment of proliferation ability. 72 h after seeding, cell proliferation was evaluated using the Cell Counting Kit-8 (Dojindo, Kumamoto, Japan). All experiments were performed in triplicate.

### In vitro tube formation assay

For in vitro tube formation assay, EPCs were seeded in the presence of EGM-2-MV medium at a density of 2 × 10^4^/mL for 8 h at 37 °C in a 48-well plate coated with Matrigel (R&D Systems, MN, USA). The formation of capillary-like structures was captured under a light microscope. Each experiment was done in triplicate.

### Luciferase assay

The 3′-UTR of FASLG containing the putative miRNA target site(s) was cloned into the SpeI and HindIII sites of the pMIR-REPORT Luciferase vector (Ambion, TX, USA). 293T cells were transfected with firefly luciferase reporter vector, miRNA, and renilla luciferase control vector using lipofectamine 3000. The reporter assays were analyzed by the examination of ratio between firefly and renilla luciferase activities. The experiments were performed in triplicate.

### Western blotting

Total proteins extracted from EPCs using RIPA buffer (Sigma-Aldrich, St. Louis, MO, USA) were separated by SDS-polyacrylamide gel and transferred into PVDF membranes. Membranes were blocked with 5% non-fat milk TBST and incubated with primary antibody for FASLG (Abcam, Cambridge, MA, USA), followed by the incubation with appropriate HRP-conjugated secondary antibody. β-actin (Sigma-Aldrich, St. Louis, MO, USA) was then measured as internal control. The densitometry of western blot results was measured using ImageJ software.

### Generation of recombinant lentivirus miR-21 and injection

The lentiviral expression vector pGLV3-H1-Puro-miR-21 was constructed to stably express miR-21 in EPCs. 293T cells were cotransfected with pGLV3-H1-Puro vector or pGLV3-H1-Puro-miR-21 plasmid using lipofectamine 3000 (Invitrogen; Carlsbad, CA, USA). Then viral particles were harvested from 293T cells and enriched. Finally, viral titers were determined by counting the labeled cells or using qRT-PCR to detect GFP expression. Lentivirus miR-21 or lentivirus vector was used to transfer miRNA into rats. Three days after thrombus formation, rats were injected within the thrombus with different solution. The solution contained 1 × 10^9^ TU/mL lentivirus miR-21 or lentiviral particles. The rats in normal group were injected with 2 mL EGM-2-MV medium.

### Histological analyses

Seven days after injection, the rats were perfused and IVC segments with thrombus were removed and fixed in 4% paraformaldehyde, embedded in paraffin. All the fixed tissue was sliced at 8-μm intervals. Hematoxylin/eosin staining were done using standard procedures. Images were captured using an inverted microscope. Before the thrombi were weighed, excessive blood on the thrombi was removed by filter paper.

### Digital subtraction angiography (DSA)

Seven days after injection, IVC venography was acquired with digital subtract angiography (DSA, GE Innova 3100, USA) by injecting contrast media into rat caudal vein or femoral vein to determine the recanalization and resolution of thrombus in vivo. All the acquired images were analyzed using image J software.

### Outcome measure

Patients attended follow-up visits at 1 month and 3 months after treatment and every 6 months thereafter and were contacted by telephone or e-mail at the 3-month mark between visits. The primary outcome of the study was either a recurrence of venous thromboembolism or PTS. Recurrent DVT was defined as a composite of symptomatic, objectively confirmed deep-vein thrombosis, nonfatal pulmonary embolism, or fatal pulmonary embolism. PTS was defined as patients with suggested symptoms including pain, heaviness, edema, varicose vein, discoloration and or ulcer in affected lower extremity. Villalta score was recorded to assess the severity of PTS during follow-up.

### Statistical analyses

Data are presented as mean ± SEM. Differences among groups were tested by one-way ANOVA. Statistical analyses between two groups were evaluated based on the Student’s two-tailed t- test. Kaplan–Meier method was used to compare the recurrent DVT between patients in different groups with log-rank test. Univariate associations between candidate predictors and recurrence of DVT were examined with 95% confidence interval (CI) by using Cox proportional hazards model. Multivariate Cox regression analysis with backward conditional method was performed to select significant prognostic factors. In all analyses, p < 0.05 was considered significant.

## Results

### Repressed expression of miR-21 in DVT model

**Fig. 1:**
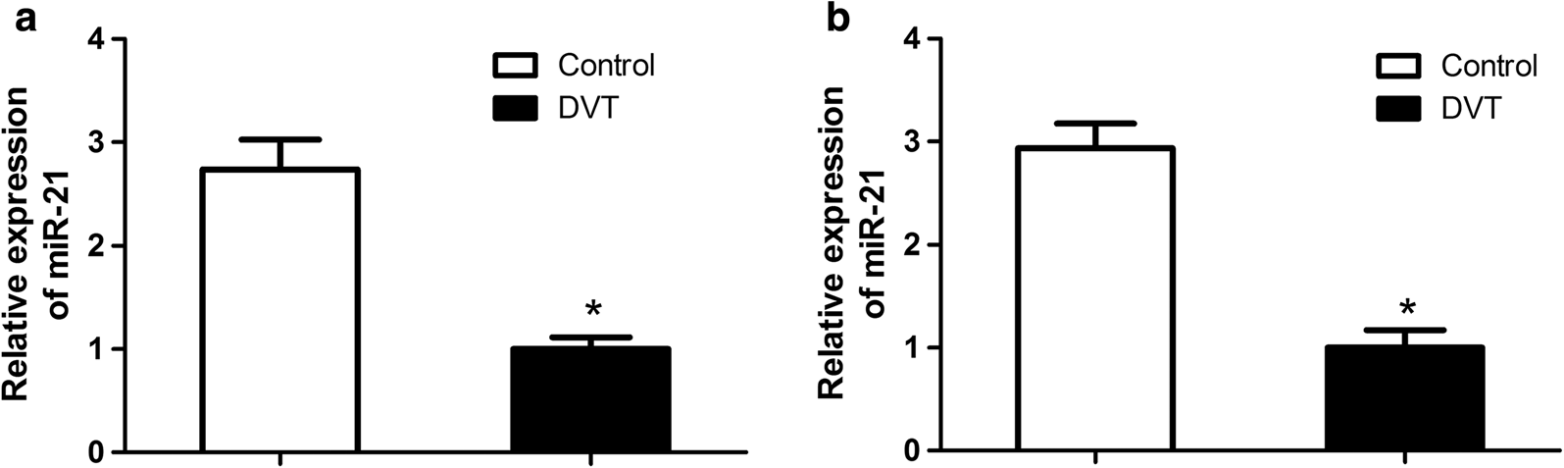
The miR-21 expression level was evaluated by quantitative real-time PCR. **a** The miR-21 expression was downregulated in DVT model compared with control group (n = 3). **b** Ten additional DVT models were included to further determined the inhibition of miR-21 expression (n = 10). *p < 0.05 vs. control group

By using endothelial progenitor cells isolated from control (n = 3) and DVT model (n = 3), we found that miR-21 was shown to be significantly down-regulated in DVT model (Fig. 1a). To further verify the downregulation trend of miR-21 in DVT model, EPCs from ten additional DVT model and control were further examined using quantitative RT-PCR. The results revealed that miR-21 had an average of 2.8-fold higher expression level in control than in DVT model (Fig. 1b).

### Upregulation of miR-21 contributes proliferation and angiogenesis of EPCs in vitro

**Fig. 2:**
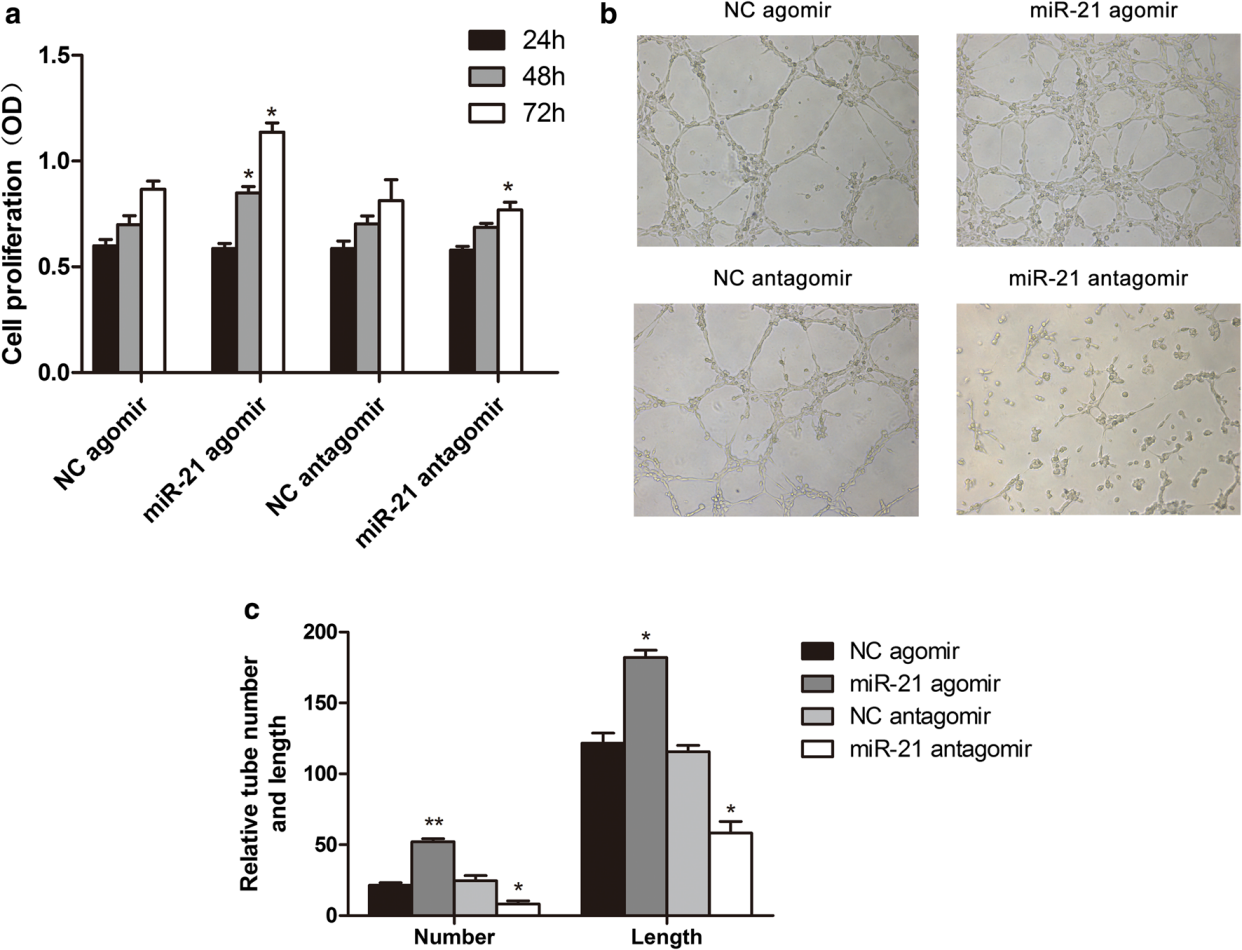
The effect of miR-21 on proliferation and angiogenesis on EPCs. **a** CCK-8 assay of proliferation of EPCs at 24, 48 and 96 h after transfection with NC agomir, miR-21 agomir, NC antagomir or miR-21 antagomir, respectively and miR-21 overexpression could increase EPCs proliferation. **b** In vitro tube formation assay was used to determine the angiogenesis ability of EPCs within different groups. MiR-21 overexpression and inhibition could increase and decrease angiogenesis in vitro, respectively. Relative tube number and length (×100) (n = 3). **c** Quantification of tube formation and length in different groups. *p < 0.05 vs. negative control

To determine the effect of miR-21 on EPCs function, we tested the proliferation and angiogenesis of EPCs transfected with miR-21 agomir or antagomir. CCK-8 assay showed that miR-21 agomir significantly promoted proliferation of EPCs compared with that in control group, whereas miR-21 antagomir decreased the proliferation of EPCs (Fig. 2a). Furthermore, we test pro-angiogenic effect of miR-21 on EPCs. As is shown in Fig. 2b, c, EPCs transfected with miR-21 agomir exhibited increased angiogenic capability when compared with those transfected with vehicle control, whereas miR-21 antagomir showed decreased angiogenic potential.

### FASLG contains a putative miR-21 binding sites

**Fig. 3:**
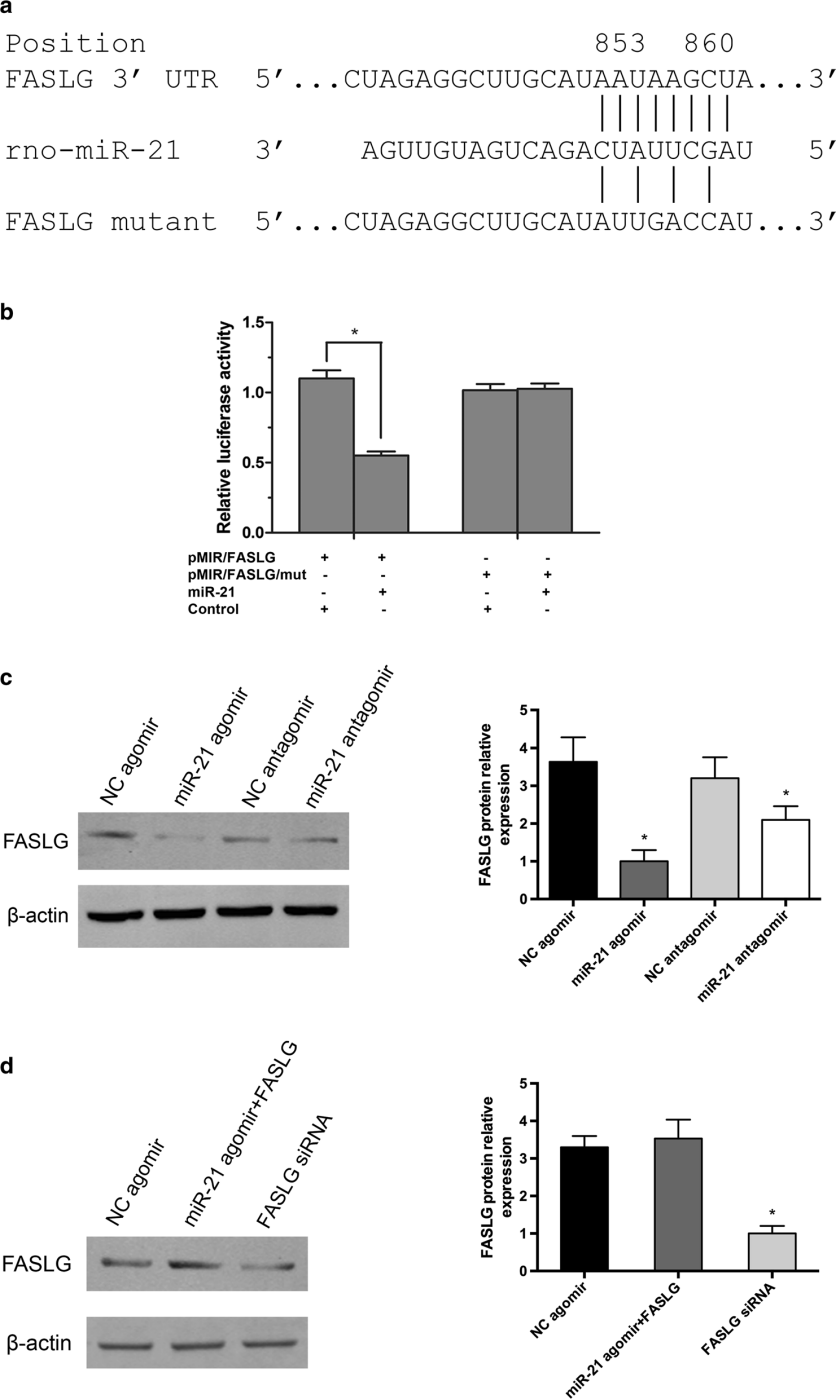
FASLG contained a potential miR-21 binding sites. **a** Sequence alignment of miR-21 with the putative binding sites in the FASLG gene, as detected by TargetScan. FASLG mutant indicated the FASLG 3′UTR with mutations in miR-21 binding region. **b** Luciferase report assays of constructs with FASLG 3′UTR or mutated FASLG 3′UTR in the absence or presence of the indicated miRNAs. **c** Western blot detection of FASLG protein in EPCs transfected with different duplexes. (Left panel) quantitative analysis of FASLG protein expression. (Right panel) **d** the protein level of FASLG in EPCs transfected with NC agomir, miR-21 agomir and FASLG, or FASLG siRNA, respectively. (Left panel) quantitative analysis of FASLG protein expression. (Right panel) β-actin served as internal control. *p < 0.05 vs. negative control

As is known to all that miRNAs cause mRNA cleavage or translational repression by forming imperfect base pairing with the 3′UTR of target genes. We searched multiple database including TargetScan, miRDB and Miranda, the results suggested that FASLG might be a potential target of miR-21, which contained a miR-21 binding site in 3′UTR region (Fig. 3a). To confirm that miR-21 could bind FASLG mRNA 3′UTR region, we constructed a luciferase reporter vector which contained this region. The results revealed that luciferase activity significantly decreased in the presence of miR-21. However, miR-21 could not affect luciferase activity if 3′UTR FASLG was muted (Fig. 3b). To further address this question, we applied miR-21 agomir and antagomir to determine the expression of FASLG protein levels. As is shown in Fig. 3c, FASLG protein level is significantly lower than that in the group of EPC transfected with miR-21 agomir, while EPCs transfected with miR-21 antagomir upregulated FASLG protein expression. Moreover, decreased FASLG protein level was observed when we employed FASLG siRNA in EPCs (Fig. 3d).

### Upregulation of miR-21 contributed to proliferation and angiogenesis of EPCs via targeting FASLG

**Fig. 4:**
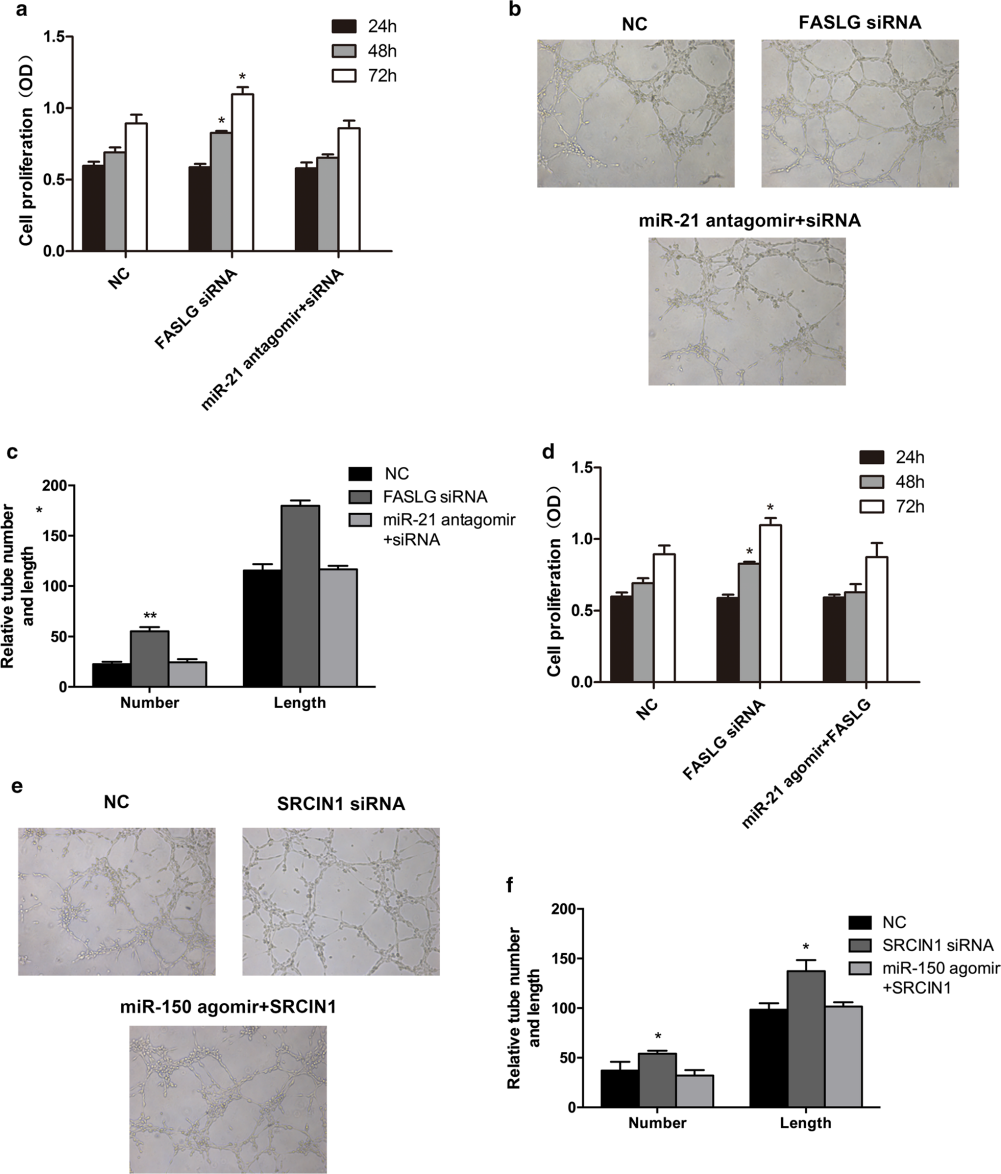
MiR-21 and FASLG regulated EPCs function in vitro. **a** CCK-8 analysis of EPCs transfected with FASLG siRNA, miR-21 antagomir and FASLG siRNA, respectively. **b** In vitro tube formation assay analysis of the angiogenesis ability of EPCs within different groups. **c** Quantification of tube formation and length in different groups. **d** CCK-8 were performed to determine the proliferation ability in EPCs co-transfected with miR-21 antagomir and FASLG siRNA. **e** Tube formation assay were performed to determine angiogenesis within different groups. **f** Quantification of tube formation and length in different groups. *p < 0.05 vs. negative control. **p < 0.01 vs. negative control

To test the effect of FASLG on EPCs function, FASLG siRNA was applied to downregulate the expression of FASLG. EPCs transfected with FASLG siRNA revealed increased cell proliferation and angiogenesis when compared to that of control group. However, miR-21 antagomir reversed this effect when co-transfected with FASLG siRNA in EPCs (Fig. 4a–c). Furthermore, we performed the rescue experiment and found the similar results when co-transfected with miR-21 agomir and FASLG (Fig. 4d–f). Taken together, these results demonstrated that miR-21 regulated function of EPCs via targeting FASLG.

### Upregulation of miR-21 increased thrombus resolution in vivo

**Fig. 5:**
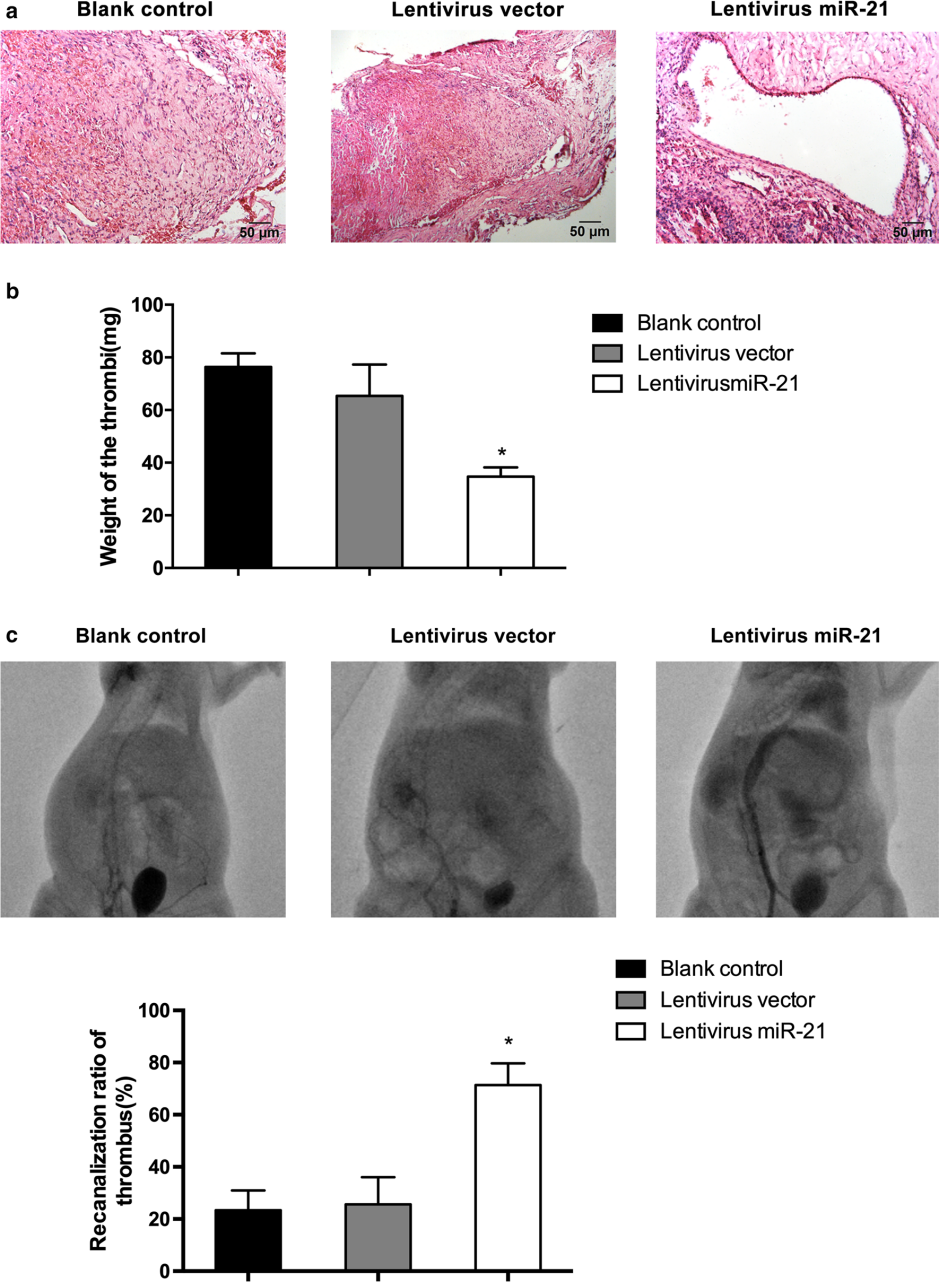
MiR-21 inhibited thrombus formation in vivo. **a** Hematoxylin and eosin staining in inferior vein cava without treatment or treated with lentivirus vector or lentivirus miR-21, respectively. **b** Weight of thrombi in IVC segment. **c** Thrombus recanalization and resolution was examined in rats transplanted with EPCs transfected with miR-21 compared to EPCs transfected vector via DSA on day 7. (Upper panel) quantitative analysis of recanalization ratio of thrombus. (Lower panel) *p < 0.05 vs. blank control

To verify whether miR-21 could affect the prognosis of DVT in vivo, a rat model of DVT was established. Cell culture medium, lentivirus miR-21 or lentivirus vector was injected 3 days after thrombus formation. Compared with blank control and vehicle control groups, the rats injected with lentivirus miR-21 showed less thrombosis in IVC segment (Fig. 5a). Moreover, in the lentivirus miR-21 groups, the weight of thrombus was obviously lower than that in other groups (Fig. 5b). Besides, we investigated the thrombus resolution in vivo by DSA. The results showed that significant increase of recanalization ratio in rats received lentivirus miR-21 compared to rats received lentivirus vector (Fig. 5c). Collectively, these results confirmed that miR-21 contributed to thrombus resolution in vivo.

### miR-21 is a prognostic marker for DVT patients

**Fig. 6:**
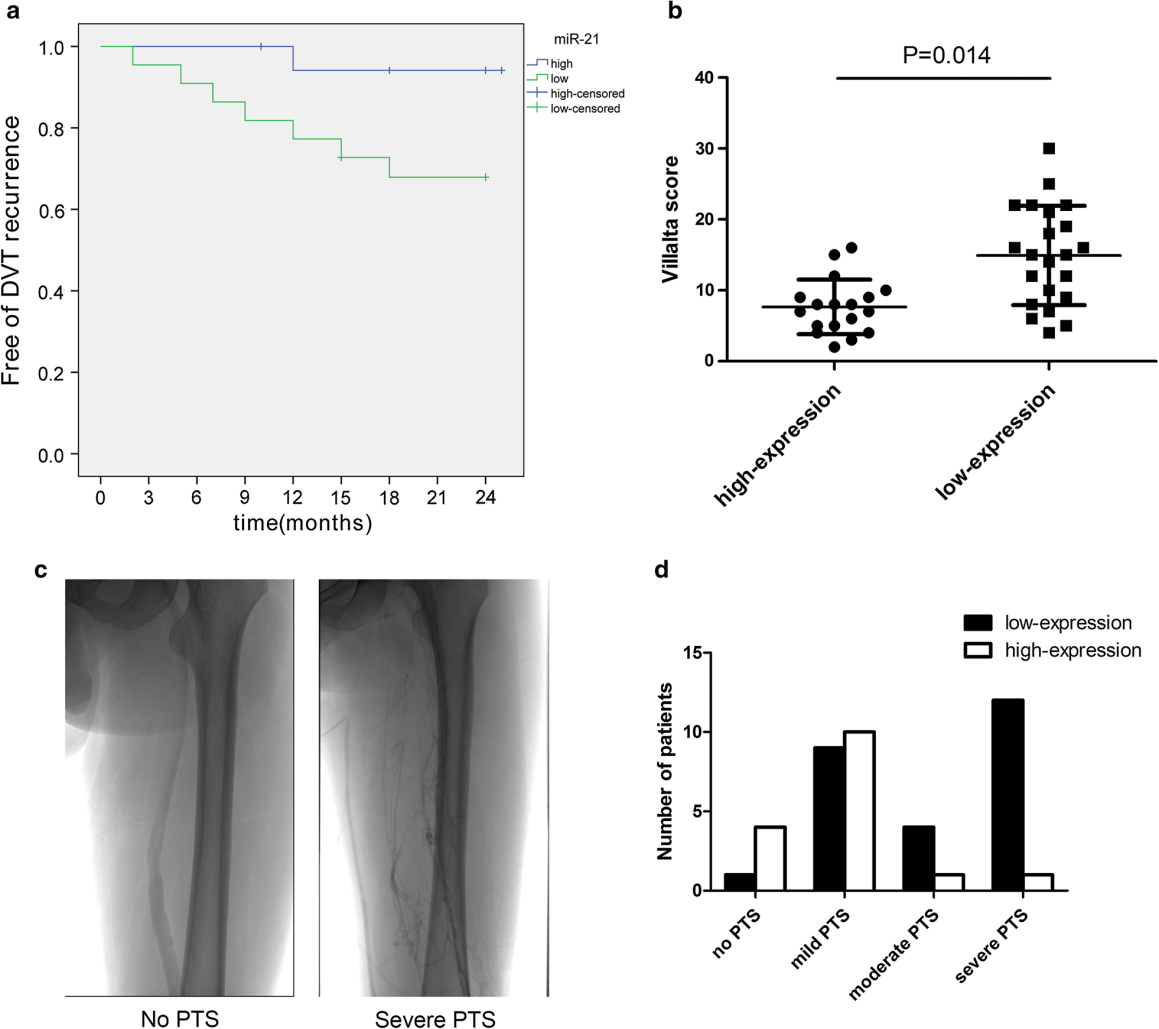
miR-21 is a prognostic marker in DVT patients. **a** Kaplan–Meier curve for recurrent DVT in a cohort of 40 DVT patients grouped by the serum level of miR-21. **b** Villalta score was evaluated in all patients grouped by the serum level of miR-21. **c** Representative images for PTS using DSA. **d** Number of PTS patients within different severity grouped by serum level of miR-21. *p < 0.05 vs. high expression group

Since miR-21 regulated EPCs biological function and promoted thrombus resolution, we then investigated miR-21 expression level in DVT patients by using RT-PCR. A total of 146 patients with DVT were referred to our department from July 2012 to June 2015. In the initial phase, 63 patients were excluded based on the exclusion criteria. In addition, 11 patients refused to provide blood sample were also excluded. Thus, we collected specimen from 72 patients. 18 patients were geographically unable to return for follow-up visits and 10 patients were confirmed with invalid Villalta score. Another four cases were excluded for case–control purpose. Finally, 40 patients were recruited in this study (Additional file 1: Figure S1). 40 patients were divided into two groups with low or high serum miR-21 using the median expression level of all 72 cases as the cut-off point. The follow‑up duration was 2 year. The patient demographic data are shown in Table 1. Kaplan–Meier analysis revealed that patients with low level of serum miR-21 had unfavorable trends of recurrent DVT. There was significant difference on recurrence-free rate between high level and low level group using the log-rank test (94.7% vs 64.6%, p = 0.019), respectively (Fig. 6a). PTS is a complication of DVT, which affect 23–60% of patients in the 2 years following DVT of the leg. PTS was assessed using the Villalta scale (Table 2), consisting of five patient‑rated leg symptoms and six clinician-rated physical signs. The results showed that patients with high miR-21 expression had lower Villalta score at 2 years after treatment (Fig. 6b). Accordingly, PTS was considered present if the Villalta score was > 4 in the leg ipsilateral to DVT and was categorized as mild if the score was 5–9, moderate if the score was 10–14 and severe if the score was > 14 or an ulcer was present. Figure 6c were representative images for PTS patients using digital subtraction angiography (DSA). We evaluate the number of patients with different severity of PTS categorized by miR-21 expression level. Our data showed that patients with more severe PTS have lower miR-21 expression level while patients with mild PTS have higher miR-21 expression level (Fig. 6d).

**Table 1:** Demographic data of patients

Characteristics	Value
Age (year, mean ± SD)	42.1 ± 8.6
Male	17 (42.5%)
Symptoms of DVT	
Leg pain	38 (95%)
Leg swelling	21 (52.5%)
Leg redness	2 (5%)
Vein of thrombosis	
Iliac vein	32 (80%)
Femoral vein	28 (70%)

**Table 2:** Villalta scale for assessment of PTS

	Absent	Mild	Moderate	Severe
Patient-rated venous symptoms				
Pain	0	1	2	3
Cramps	0	1	2	3
Heaviness	0	1	2	3
Paraesthesia	0	1	2	3
Pruritus	0	1	2	3
Clinician-rated signs				
Pretibial edema	0	1	2	3
Skin induration	0	1	2	3
Hyperpigmentation	0	1	2	3
Pain during calf compression	0	1	2	3
Venous ectasia	0	1	2	3
Redness	0	1	2	3

Each symptom/sign rated as 0 (absent), 1 (mild), 2 (moderate) or 3 (severe); all numeric points are summed to yield a total score: PTS is considered absent if the Villalta score is < 4; mild PTS, 5–9; moderate PTS, 10–14; and severe PTS if the score is ≥ 14 or an ulcer is present

### miR-21 independently predicts the recurrence of DVT

To further investigate independent risk factors for recurrence of DVT. Six candidate predictors including age, gender, BMI (BMI ≥ 30 or BMI < 30), categories of DVT (unprovoked or secondary to risk factors), duration of anticoagulation and miR-21 expression (low level or high level) were selected based on existing retrospective researches and a priori hypotheses. Univariate Cox proportional hazard regression analyses were performed and found that miR-21 expression level was significantly associated with recurrence of DVT. The other significant prognostic factors in univariate analyses including age, category of DVT, duration of anticoagulation and miR-21 expression (Table 3). Moreover, multivariate analysis indicated that miR-21 expression, age and duration of anticoagulation demonstrated significant correlation with recurrence (Table 3). Taken together, these results showed that miR-21 expression level can serve as an independent predictor of recurrence of DVT.

**Table 3:** Univariate and multivariate Cox regression analysis of recurrence of DVT

Characteristics	Univariate analysis				Multivariate analysis	
	HR	95% CI	p value	HR	95% CI	p value
Age	1.160	1.011–1.330	0.021	1.270	1.012–1.593	0.039
Gender	0.674	0.161–2.822	0.584	–	–	–
BMI (≥ 30)	1.264	0.316–5.055	0.741	–	–	–
Category of DVT (secondary)	4.435	1.489–22.028	0.047	1.741	0.290–10.451	0.554
Duration of anticoagulation	0.905	0.831–0.985	0.014	0.894	0.824–0.971	0.008
miR-21 (high)	0.125	0.015–0.920	0.015	0.085	0.009–0.768	0.028

## Discussion

In our present study, we find repressed expression of miR-21 in EPCs from DVT rats. Furthermore, we determined that upregulation of miR-21 expression promoted angiogenesis and proliferation of EPCs. In addition, we confirmed that miR-21 regulated function of EPCs via targeting FASLG, thus upregulation of miR-21 expression leading to increased thrombus resolution in a murine model of venous thrombosis. Finally, lower expression level of miR-21 in DVT patients was associated with an increase of recurrent DVT and severe PTS.

Thrombosis is the formation of occlusive blood clot inside a blood vessel, causing obstruction of blood flow and reduced nutrients and oxygen delivery to distal tissue. Finally, thrombosis may lead to tissue and organ necrosis. The endothelium serves as integral role in the hemostatic system and regarded as a barrier which separates the blood from the surrounding tissue. Normal endothelial cells could express anticoagulant molecules that inhibit platelet aggregation and fibrin formation [18]. In addition, endothelial cells contribute to tissue repair and angiogenesis in face of stable clot. Thus functional endothelial cells are important for prevention of thrombosis. Endothelial progenitor cells are the precursor cells of mature endothelial cells, which play an important role in pathological and physiological neovascularization in the adult [19, 20].

MicroRNA are a large family consisting of ~ 21-nucleotide-long RNAs, which plays an important role in regulating post-transcriptional gene expression [21, 22]. It has been demonstrated that miRNA are involved in multiple biological processes including vascular development, homeostasis and differentiation [23]. The biological roles of miR-21 are well demonstrated in tumor studies. Previous study has revealed that expression of miR-21 was highly elevated in breast cancer and inhibition of miR-21 down-regulated apoptosis and cell proliferation in tumor cells [24]. Chan et al. [25] found that expression of miR-21 was significantly elevated in human glioblastoma. Recently, miR-21 has been found to be highly expressed in main types of vascular cells, including vascular smooth muscle cell (VSMC) [26] and endothelial cell [27]. For example, Wang et al. [28] reported miR-21 regulated vascular smooth muscle cell function via targeting tropomyosin 1 in arteriosclerosis obliterans (ASO) of lower extremities. A study from Ji et al. [26] showed that knock-down of the overexpressed miR-21 in balloon-injured rat carotid arteries inhibited neointimal lesion growth significantly. Indeed, miR-21 plays important roles in proliferative arterial disorders. However, the effect of miR-21 on the function of EPCs and its association with venous thrombosis are little known. Liu et al. [29] demonstrated that miR-21 promoted angiogenesis of tumor endothelial cells through targeting PTEN, leading to activate AKT and ERK1/2 signaling pathways, and thereby enhancing HIF-1α and VEGF expression. In the current study, we conducted ex vivo experiments to identify the effect of miR-21 on angiogenesis and proliferation of endothelial progenitor cells. The results showed that miR-21 contributed endothelial progenitor cell angiogenesis and proliferation. Besides, miR-21 was repressed in progenitor endothelial cells under DVT and upregulation of miR-21 promoted thrombus resolution in vivo, suggesting it might be a therapeutic target for treatment of DVT.

FASLG and its receptor FAS are members of tumor necrosis factor (TNF) superfamily. FASLG has been regarded as an important effector involving in various biological events. For example, the binding of membrane-bound FASLG to Fas expressing cells was associated with cell death via activating pro-apoptotic signaling cascade [30]. Furthermore, several studies claimed that decreased FASLG expression affected cell apoptosis and proliferation among different cancers such as breast and colorectal cancer and hepatocellular [31–33]. Interestingly, bioinformatic analysis revealed that FASLG contained a putative conserved binding region for miR-21. So we further explore the potential correlation of FASLG and miR-21 in regulating the function of EPCs. Our results showed that miR-21 could inhibit FASLG expression eventually promoting EPCs proliferation and angiogenesis.

Previous study has demonstrated that miRNAs could be stably detected in plasma which could contribute to the detection of prostate cancer [34], many investigators have identified specific miRNAs for screening and predicting prognosis of various diseases. Zhou et al. [35] reported MiR-28-3p as a potential plasma marker in diagnosis of pulmonary embolism. Xiao et al. [36] previously found that increased plasma miR-134 could distinguish PE patients from normal individuals, and miR-210 was proved to be hypoxia-associated. Here, we found that DVT patients with low level of serum miR-21 had unfavorable trends of recurrent risk. Also, patients with more severe PTS have lower miR-21 expression level.

## Conclusions

In summary, our studies have found that miR-21 expression was repressed in DVT model and it played a critical role in regulating proliferation and angiogenesis of EPCs via targeting FASLG. In addition, injection of overexpressed miR-21 in thrombosis segment promoted thrombus resolution in vivo. Lower expression level of miR-21 in DVT patients was associated with severe PTS and could be an independent risk factor in predicting recurrence of DVT. Taken together, miR-21 could be a novel therapeutic target in the treatment of thrombus in the clinical practice.

## Additional file

**Additional file 1: Figure** **S1.** Flow chart of the patients included in this study.

## Data Availability

The datasets used and/or analyzed during the current study are available from the corresponding author on reasonable request.

## Abbreviations

DVT: deep venous thrombosis
EPC: endothelial progenitor cells
miRNA: microRNA
PTS: post thrombotic syndrome
HUVECs: human umbilical vein endothelial cells
SPF: specific pathogen-free
IVC: inferior vena cava
