# Supplementary figures and images for: miR-21 induces endothelial progenitor cells proliferation and angiogenesis via targeting FASLG and is a potential prognostic marker in deep venous thrombosis
Source: J Transl Med. 2019 Aug 15;17:270. doi: 10.1186/s12967-019-2015-z (PMC6694687; doi:10.1186/s12967-019-2015-z)

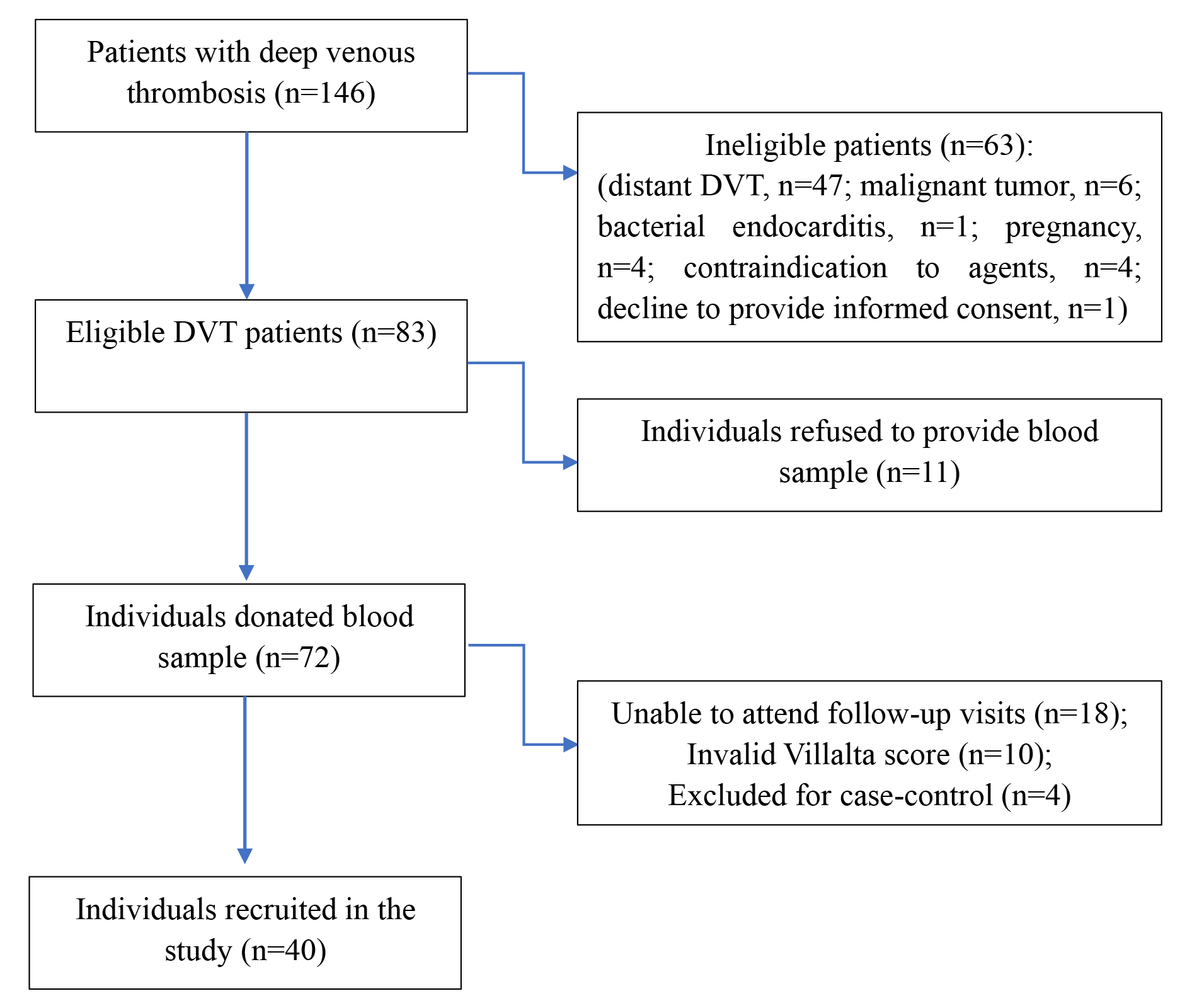

Supplement: Supplementary file 1 — Additional file 1: Figure S1. Flow chart of the patients included in this study. [file 12967_2019_2015_MOESM1_ESM.tif]
